# Molecular identification and biological characterization of *Cryptosporidium muris* from camels (*Camelus bactrianus*) in China

**DOI:** 10.1186/s13071-021-04862-8

**Published:** 2021-07-15

**Authors:** Luyang Wang, Letian Cao, Shuangjian Zheng, Yankai Chang, Kaihui Zhang, Sumei Zhang, Longxian Zhang

**Affiliations:** 1 College of Veterinary Medicine, Henan Agricultural University, Zhengzhou, Henan People’s Republic of China; 2 International Joint Research Laboratory for Zoonotic Diseases of Henan, Zhengzhou, Henan People’s Republic of China

**Keywords:** *Cryptosporidium muris*, Bactrian camel, Multilocus analysis, MLST subtype, Experimental infection model

## Abstract

**Background:**

*Cryptosporidium* is an opportunistic pathogen that infects a wide variety of vertebrates. The aim of the present study was to characterize *Cryptosporidium* spp. isolates from Bactrian camels and to foster further understanding of the biological characteristics of the pathogen.

**Methods:**

Fecal specimens were collected from two 4-year-old Bactrian camels resident at the Kaifeng City Zoo in China and examined for *Cryptosporidium*. Fecal specimens were screened using the floatation method, and then genomic DNA was extracted from the oocysts and identified by nested-PCR amplification of the small subunit ribosomal RNA (SSU rRNA) gene, the actin gene and the *Cryptosporidium* oocyst wall-protein (COWP) gene. Subtype analysis was performed based on four minisatellite (MS) loci (MS1, MS2, MS3 and MS16) that were aligned and phylogenetically analyzed to determine the species and subtype of *Cryptosporidium*. We then established a BALB/c mice infection model and further verified the results through clinical status, pattern of oocyst excretion and histological examination.

**Results:**

*Cryptosporidium* oocyst isolates from the two Bactrian camels had an average (± standard deviation) size of 7.49 ± 0.13 × 5.70 ± 0.10 μm (*n* = 50). The sequencing and phylogenetic analysis confirmed the species as *C. muris*. Multilocus sequence typing analysis indicated that the subtypes were M13, M4, M1 and M5. Following the inoculation of BALB/c mice, we found that the prepatent period and number of oocysts per gram increased with increasing infective dose. Oocysts were first detected in the feces of BALB/c mice at 7–8 days post-infection (dpi), with levels peaking twice thereafter, at 15–16 dpi and 19–20 dpi. Histology and scanning electron microscopy studies showed that the stomach contained gastric pits filled with *Cryptosporidium* that adhered to the surface of gastric mucosa gland epithelial cells, causing the latter to deform, swell and become disordered.

**Conclusions:**

The findings of this study indicated that oocysts isolated from Bactrian camels were from *C. muris*. This is the first report of *C. muris* isolated from camels in China. More epidemiological data are needed to understand the prevalence and transmission of *C. muris* in camels in different geographic areas.

**Graphical abstract:**

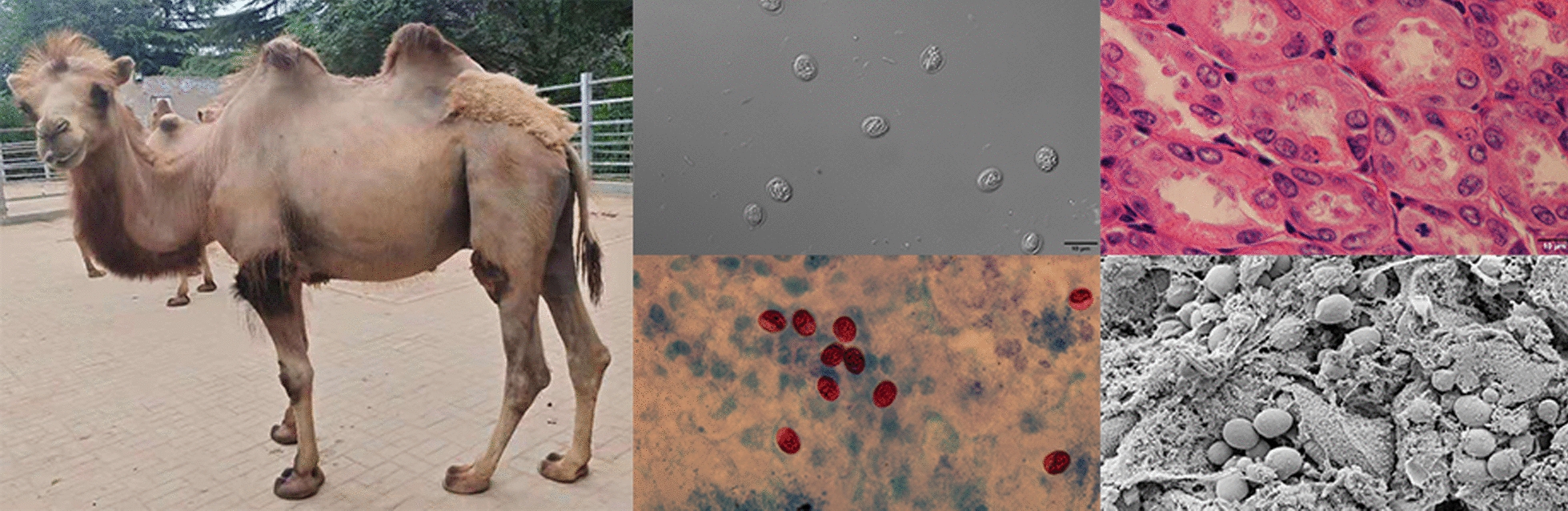

## Background

Cryptosporidiosis is a common and widely distributed zoonotic disease of significant public health concern worldwide that is caused by a species of the genus *Cryptosporidium*. The parasite has multiple vertebrate hosts, including humans, and cryptosporidiosis is one of the most common causes of diarrheal diseases in humans and domestic animals [1, 2]. According to molecular analyses and morphological data, 46 *Cryptosporidium* species and > 100 *Cryptosporidium* genotypes have been identified so far in various hosts [3].

Molecular diagnostic methods have long been an important tool for studying the transmission of *Cryptosporidium* spp. [4]. One of the drivers of this strategy is that different *Cryptosporidium* species and genotypes have a certain degree of morphological similarity, making identification by conventional microscopic examination difficult. This difficulty has resulted in the development of a nested-PCR molecular diagnostic tool based on polymorphism of the small ribosomal subunit (*SSU*) gene. *Cryptosporidium muris* and *C. andersoni* are the most common gastric *Cryptosporidium* species. Due to the strong similarity between these two species in terms of their genetics and biology, researchers have developed a multiocus sequence typing (MLST) tool using polymorphic microsatellites and microsatellite repeat markers to distinguish between them [5]. This method has been used effectively for characterizing the population genetics of *C. muris* and *C. andersoni* [6].

In 1907, Ernest Edward Tyzzer discovered a parasite in the gastric glands of laboratory mice and named it *Cryptosporidium muris* [7]. The parasite was able to infect a variety of mammals, including various rodent species [8, 9], cats, dogs [10], crab-eating macaques [11], deer [12], horses [13], mountain goats, Bactrian camels and humans [14]. Studies conducted on the life-cycle of *C. muris* found that all stages were localized in the gastric glands of the stomach [15]. An ostrich-derived *C. muris* model of experimental infection has been developed to study cross-transmission [16]. However, cryptosporidiosis caused by other animal-derived *C. muris* infection has not yet been reported.

In this study, Bactrian camel (*Camelus bactrianus*) stool samples were diagnosed to be *Cryptosporidium* positive, and we examined oocyst morphology and molecular characteristics of this *Cryptosporidium* isolate from the camels. To further understand the pathogenicity and infectivity of this *Cryptosporidium* isolate, we developed a mouse infection model that provides a foundation for future testing of novel drugs or vaccines against cryptosporidiosis.

## Methods

### Source of oocysts

Fecal specimens from two 4-year-old female Bactrian camels were collected in December 2018 from the Kaifeng City Zoo in China. The *Cryptosporidium*-positive specimens were evaluated for the presence of *Cryptosporidium* oocysts by Sheather’s sugar floatation method [17]. We collected the *Cryptosporidium* oocysts from the feces using the water/ether concentration method [18] and purified the samples by discontinuous sucrose density centrifugation [19]. Oocysts were counted with a Neubauer hemocytometer. Micrographs of the purified *Cryptosporidium* oocysts were obtained using differential interference contrast microscopy. A combination of streptomycin and penicillin was added, and the oocysts were stored in phosphate-buffered saline (PBS) at 4 °C.

Morphometric analyses of *Cryptosporidium* oocysts were performed using an image digital analysis method (Motic Images Plus 2.0 software). The oocysts (*n* = 50) were randomly selected under an Olympus microscope at 1000-fold magnification, and their lengths and widths were measured [20].

### DNA extraction and sequence analysis

Genomic DNA was extracted from oocysts using commercial E.Z.N.A Stool DNA kits (Omega Bio-Tek Inc., Norcross, GA, USA). The oocysts were first suspended in lysis buffer and kept on ice for 30 min, then frozen in liquid nitrogen for 3 min before thawing in a 37 °C water bath; the process was repeated six times. Extracted DNA samples were stored at − 20 °C until PCR analysis.

The DNA samples were analyzed by nested-PCR amplification of the SSU rRNA gene, the actin gene and the *Cryptosporidium* oocyst wall-protein gene (COWP gene) following previously published protocols [21–24], to determine the species of the *Cryptosporidium* isolates in this study. For the SSU fragment, the external PCR primers were 5′-TTCTAGAGCTAATACATGCG-3′ and 5′-CCCATTTCCTTCGAAACAGGA-3′; the internal PCR primers were 5′-GGAAGGGTTGTATTTATTAGATAAAG-3′ and 5′-AAGGAGTAAGGAACAACCTCCA-3′. For the actin fragment, the external PCR primers were 5′-ATGCCVGGWRTWATGGTDGGTATG-3′ and 5′-GGDGCAACRACYTTRATCTTC-3′; the internal PCR primers were 5′-GAYGARGCHCARTCVAARAGRGGTAT-3′ and 5′-TTDATYTTCATDGTHGAHGGWGC-3′. For the COWP fragment, the external PCR primers were 5′-GAATGTCCTCCTGGGACTGTA-3′ and 5′-AGTTCCTGGTGGACATACTG-3′; the internal PCR primers were 5′-TCCTCCTGGGACTGTATTGG-3′ and 5′-GGTGGACATACTGGTTGTGTTG-3′. Subtype identification and analysis were performed based on four* C. muris* (CM) minisatellite (MS) gene loci: CM-MS1, CM-MS2, CM-MS3 and CM-MS16 [5, 25]. The primers and amplification conditions used in MLST subtype analysis of MS1 (coding for the hypothetical protein CMU_036310), MS2 (coding for heat shock protein), MS3 (coding for the hypothetical protein CMU_020660) and MS16 (coding for the leucine-rich repeat family protein CMU_035650) genes were as previously described [5]. The nucleotide sequences of the partial SSU rRNA, actin, COWP and MLST subtype genes of *C. muris* from the Bactrian camels were deposited in the GenBank database under accession numbers MT708027 and MT721871–MT721876.

### Phylogenetic analysis

The secondary PCR products were sent to Zhengzhou QingCheng Biotechnology Co. Ltd. for double-end sequencing (ABI PRISM 3730 XL DNA Analyzer; Applied Biosystems, Thermo Fisher Scientific, Foster City, CA, USA ). The sequencing results were corrected according to the sequencing map. After the splicing was completed, a homology sequence search was performed on GenBank, and the software Clustal X 2.13 (http://www.clustal.org/) was used for comparison and analysis to determine *Cryptosporidium* species. Then MLST was used for subtype identification.

In this study, Mega 7.0 (https://www.megasoftware.net) software was used to build evolutionary trees for different gene loci, and the neighbor-joining algorithm was used to conduct a phylogenetic analysis with 1000 bootstrap replicates based on evolutionary distances calculated by the Kimura two-parameter model.

### Experimental design

#### Experimental animals

Fifteen 4-week-old female BALB/c mice were obtained from Henan Experimental Animal Center, Zhengzhou, China. All mice were housed individually in plastic cages with wire mesh tops under pathogen-free conditions, kept on daily 12/12-h light/dark cycles and supplied with sterilized food and water. The plastic cages were disinfected with pressurized steam before use. Mice were randomly divided into a control group and two test groups, with five mice per group. In order to know whether there were differences in oocyst excretion at different doses of infection, each mouse in the two test groups was orally inoculated by stomach tube with either 1 × 10^6^ or 3 × 10^6^ *Cryptosporidium* oocysts suspended in 200 μl of distilled water. Each mouse in the control group was inoculated with an equal dose of distilled water. All experimental procedures complied with the recommendations of the Guide for the Care and Use of Laboratory Animals of the Ministry of Health, China.

#### Clinical status

Shape and color of fecal matter and clinical status of the mice were observed daily for each group sample and for each mouse, respectively. Rectal temperature, breathing, appetite and presence of any abnormal behaviors were recorded.

#### Oocyst excretion

Fresh fecal samples from each group of mice were collected daily after infection to determine the prepatent period (malachite green stain), and the experiments were terminated when no *Cryptosporidium* oocysts could be detected under the microscope. *Cryptosporidium* oocysts were isolated from fecal samples using Sheather’s sugar floatation method [17], and the number of oocysts per gram (OPG) of feces was counted using a hemocytometer slide. Each group sampling was repeated three times to calculate an average value. Number of OPG was estimated according to the number of oocysts counted and a curve diagram of oocyst excretion [26].

#### Histological examination

For histological analysis, one mouse in each group was euthanized by cervical dislocation at day 14 post infection (dpi), and tissue samples harvested from the duodenum, jejunum, ileum, cecum, stomach, lungs, liver, spleen and kidney were fixed in 10% neutral buffered formalin for 24 h at room temperature as described previously [27]. After being dehydrated in different concentrations of ethanol and cleared in xylene, the tissues were embedded in paraffin and then sectioned and stained with hematoxylin and eosin (H&E). The stained slides were observed under a microscopte at 400× and 1000× magnification [28].

#### Scanning electron microscopy

In order to better observe and further confirm the colonization of *Cryptosporidium* in mice, stomach tissue samples from mice in the two treatment groups were observed by scanning electron microscopy (SEM) based on the histological observation results. The samples were first fixed in 2.5% glutaraldehyde at 4 °C for 1 week, followed by dehydration in an ethanol gradient series (30, 50, 70, 80 and 90% ethanol) and two additional changes in 100% ethanol, each for 15 min. This was followed by immersion in 100% isoamyl acetate solution twice for 20 min. The samples were critical point-dried using CO_2_, coated with gold and observed using a scanning electron microscope (Qunata FEG 250, FEI Co., Thermo Fisher Scientific, Hillsboro, OR, USA) [20, 27].

### Statistical analysis

Differences in oocyst sizes were tested using the chi-square test in SPSS (release 13.0 standard version; SPSS-IBM Corp., Armonk, NY, USA), and differences were considered to be statistically significant at *p* < 0.05.

## Results

### Morphological measurements

**Fig. 1:**
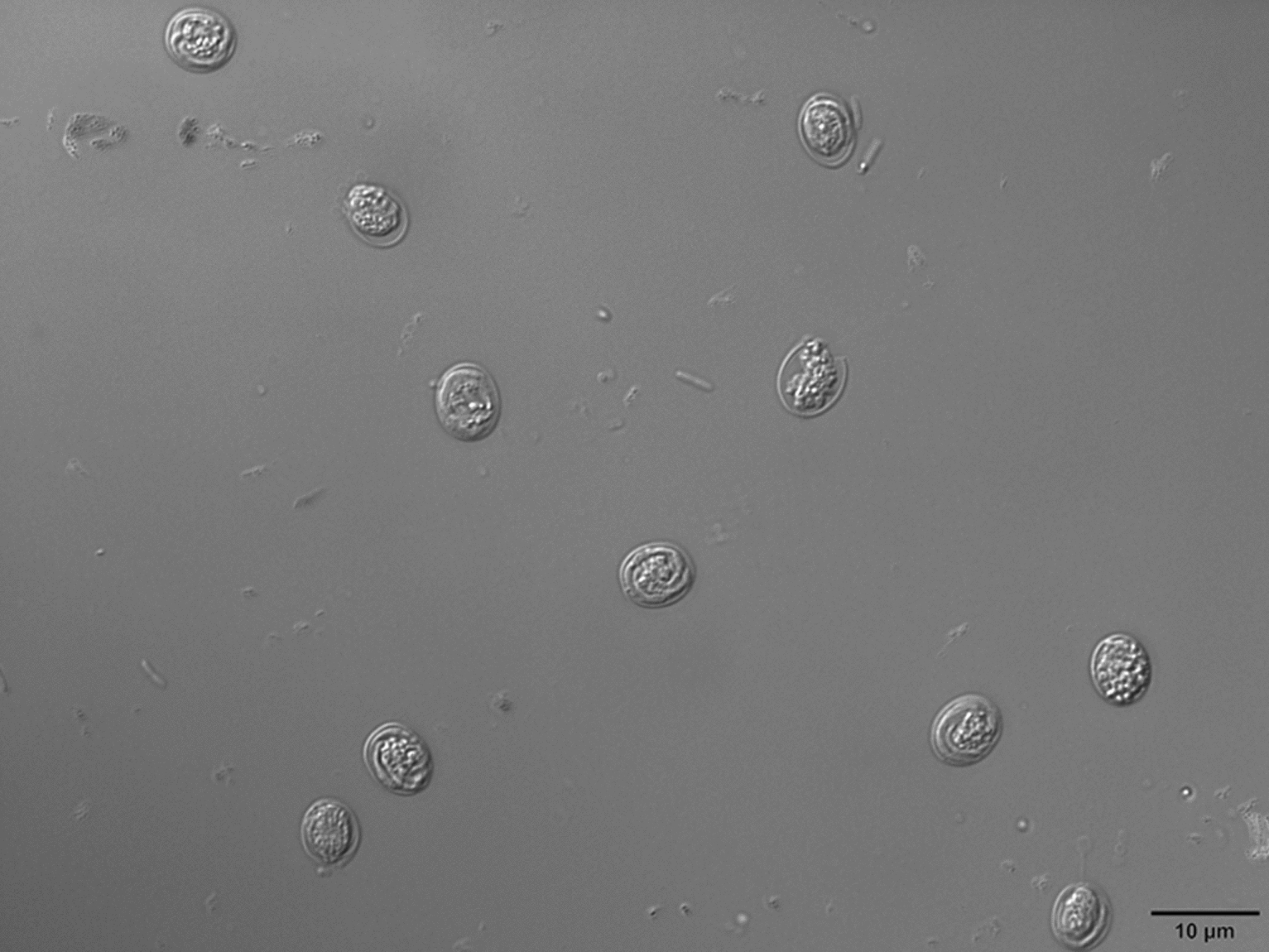
*Cryptosporidium muris* oocysts from Bactrian camels examined under differential interference contrast microscopy settings. Scale bars: 10 μm

The oocysts of *Cryptosporidium* were ellipsoidal and lacked sporocysts. The mean size (± standard deviation) of the oocysts was 7.49 ± 0.13 × 5.70 ± 0.10 μm, with a length/width ratio of 1.32 (*n* = 50). The micrographs of the purified *Cryptosporidium* oocyst are shown in Fig. 1.

### Molecular characterization

**Fig. 2:**
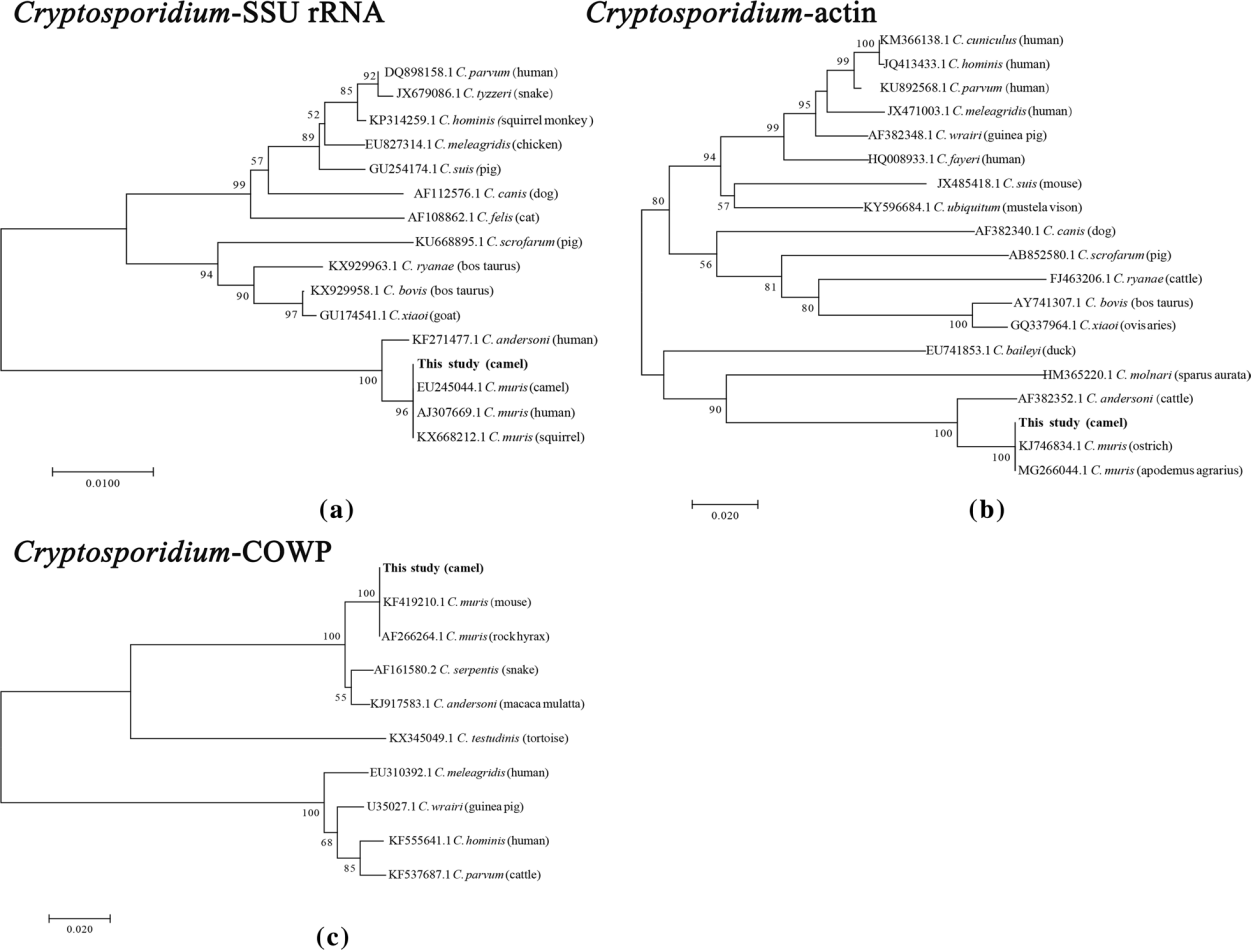
Phylogenetic relationships of *Cryptosporidium* parasites inferred by neighbor-joining analysis of the* 18S* rRNA (**a**) actin genes (**b**) and *Cryptosporidium* oocyst wall protein (COWP; **c**) based on the Kimura two-parameter model

**Fig. 3:**
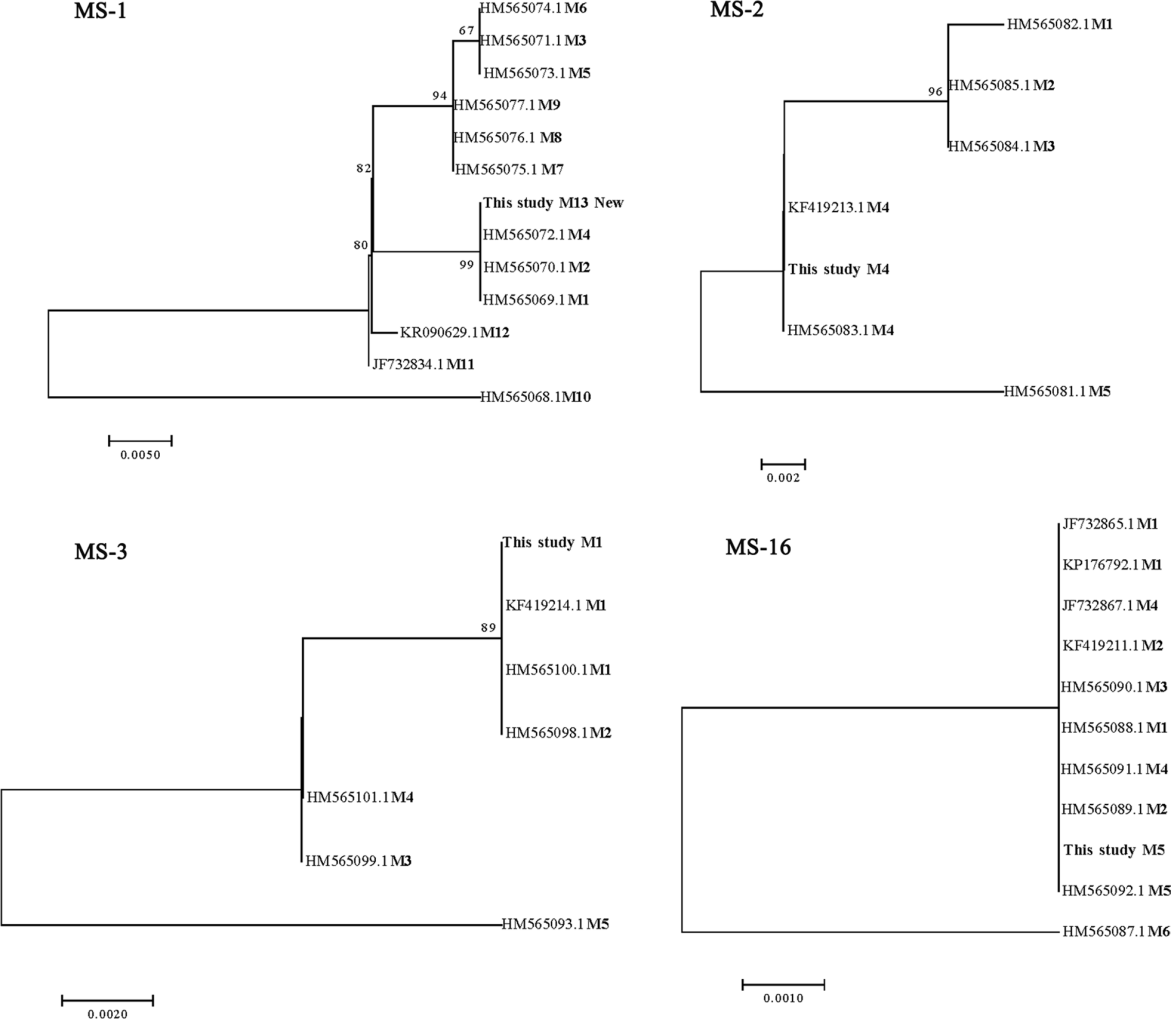
Phylogenetic relationships among subtypes of camel-derived *C. muris* isolates at four microsatellite and minisatellite (*MS*) loci (MS1, MS2, MS3, and MS16) as assessed by a neighbor-joining analysis of the nucleotide sequences, using distance calculated by the Kimura two-parameter model

For each of the gene targets, sequences of the *Cryptosporidium* isolates were aligned with those from other *C. muris* isolates using Clustal X 2.13 [29]. DNA sequencing indicated that the SSU rRNA nucleotide sequences had 96% similarity with those of *C. muris* (GenBank accession number EU245044 in the Czech Republic and AJ307669 in Kenya). The isolates formed a cluster with *C. muris* (CM), while the COWP and actin nucleotide sequences had 100% similarity with those of *C. muris* (GenBank accession numbers KF419210 and KJ746834 in China, respectively) (Fig. 2a–c). Subtype identification of this *C. muris* isolate was successfully performed at all four loci; as the CM-MS1 locus had significant differences, it was named as a new subtype: M13. The CM-MS16 locus base sequence differed by only one base and, therefore, the *C. muris* isolate formed a new *C. muris* MLST subtype: M13, M4, M1, M5 (Fig. 3).

### Clinical status of mice

The appetites and attitudes of all animals in both the test and control groups were normal during the experiment. All animals remained free of clinical signs, without changes in fecal traits, appetite, hair quality, or behavior. No remarkable changes were observed in macroscopic observations, and no diarrhea was observed in any infected animal. In addition, no animals died during the experiment.

### Oocyst shedding in infected mice

**Fig. 4:**
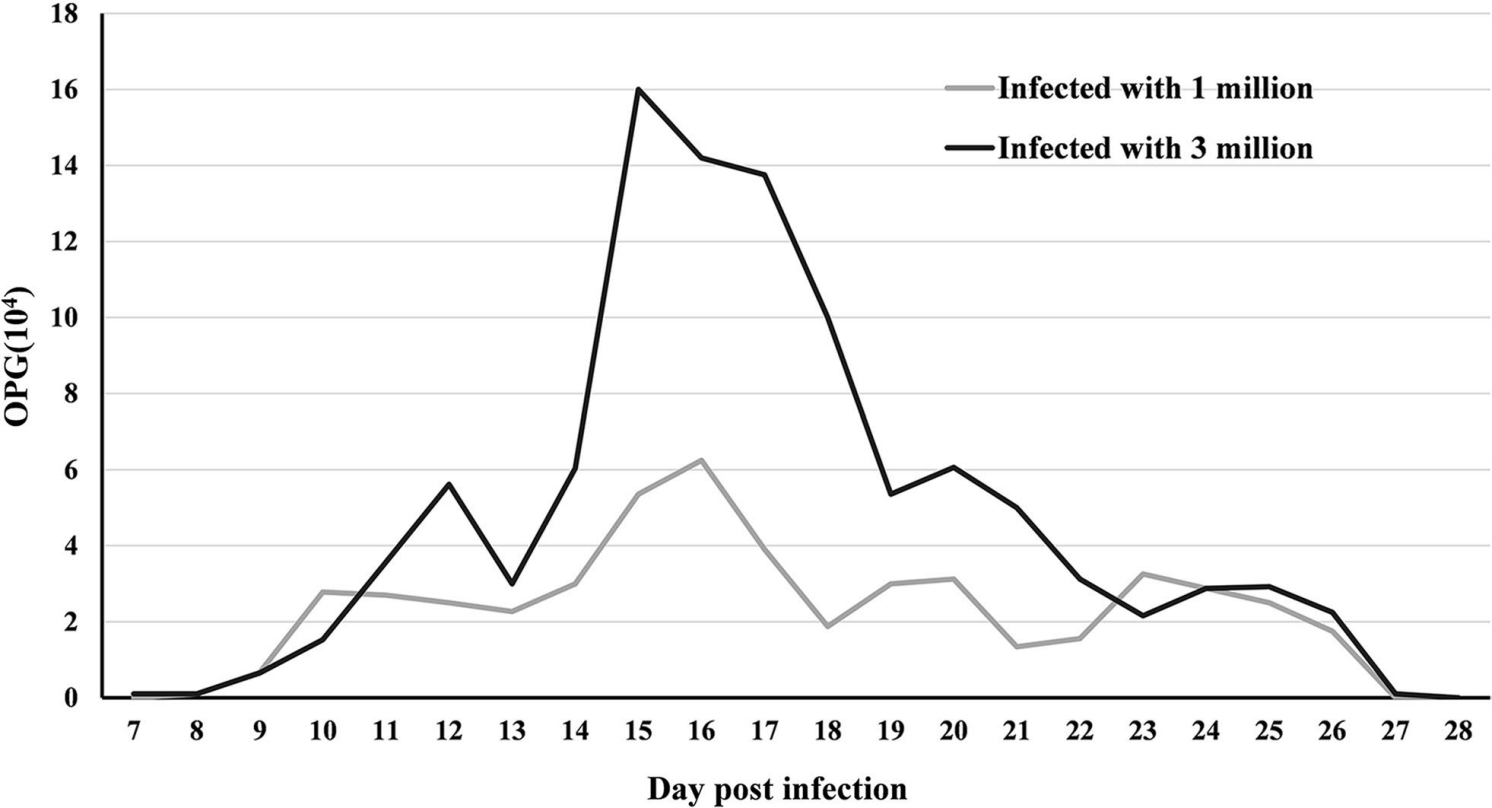
Excretion of oocysts in 1 g of feces in all mice infected with 1 × 10^6^ *C. muris* oocysts and infected with 3 × 10^6^ *C. muris* oocysts during the experiment. Arithmetic means of all the examined animals are shown. The gray line indicates the group of mice infected with 1 × 10^6^ *C. muris* oocysts. The black line indicates the group of mice infected with 3 × 10^6^ *C. muris* oocysts.* OPG* Oocytes per gram

Fecal examination showed that *Cryptosporidium* oocysts were found only in the feces of mice inoculated with *Cryptosporidium* oocysts. No *Cryptosporidium* oocysts were found in control animals during the experiment. Oocysts were first detected in feces of the two infected groups by light microscopy on 7 and 8 dpi, peaking twice on 15 and 16 dpi; all animals remained infected until 27 dpi (Fig. 4). The infection intensity of the group infected with 3 × 10^6^ oocysts ranged from 1000 to 160,000 OPG, with maximum shedding at 15 dpi, and the infection intensity of the group infected with 1 × 10^6^ oocysts ranged from 1000 to 62,500 OPG with maximum shedding at 16 dpi. The group infected with 3 × 10^6^ oocysts entered the prepatent and peak periods earlier than the group infected with 1 × 10^6^ oocysts. Both infected groups had no detectable *Cryptosporidium* oocysts in feces after 27 dpi. The patterns of oocyst shedding in mice in the infected groups are shown in Fig. 4.

### Histological observations

**Fig. 5:**
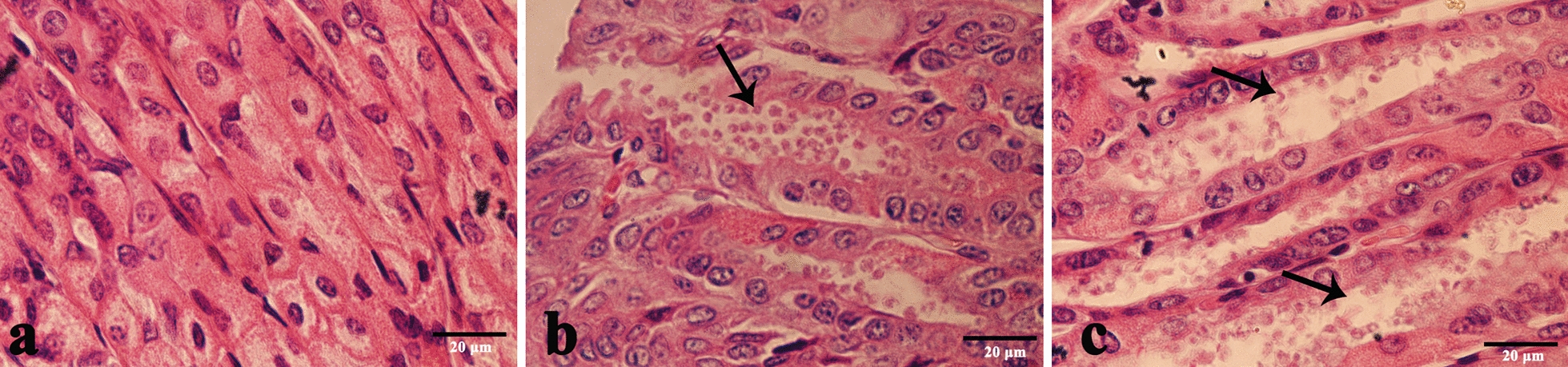
Observations of pathological sections of stomach tissue of BALB/c mice stained with hematoxylin and eosin. **a** Longitudinal section of gastric epithelium, negative control.** b**,** c** Gastric pits (arrow) in stomach tissue sections of BALB/c mice infected with 1 × 10^6^ oocysts (**b**) and 3 × 10^6^ oocysts (**c**) of *C. muris* (arrow) which were sacrificed 14 days post-infection. A scale bar is included in each figure

Histological examination of the tissue sections showed that *Cryptosporidium* parasitized the stomach tissues of infected mice (Fig. 5). No *Cryptosporidium* developmental stages or pathological changes were observed at any other anatomical site, including the duodenum, jejunum, ileum, cecum, lungs, liver, spleen and kidney; these findings were consistent for all infected mice in the study. Likewise, no *Cryptosporidium* developmental stages or pathological changes were observed at any anatomical site in mice of the control group (Fig. 5a). In the two treatment groups, the histopathological changes of the stomach included opened and enlarged gastric pits that were filled with necrotic material, mucus and numerous parasite stages. The majority of the gastric glandular parts were clearly affected; the pathological changes in the group infected with 3 × 10^6^ oocysts were more serious, and the changes included the extension of gastric longitudinal folds, epithelial hyperplasia and mucosal hypertrophy (Fig. 5b, c), while the non-glandular parts were unchanged.

### Scanning electron microscopy

**Fig. 6:**
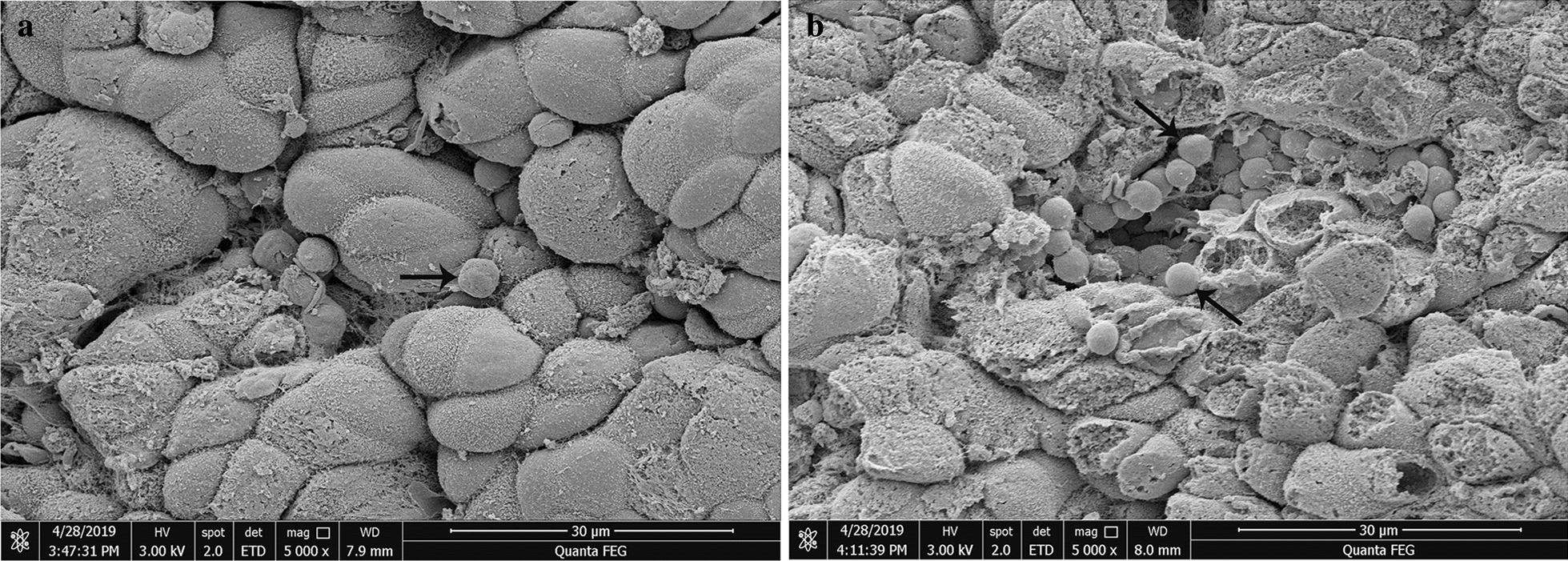
Scanning electron microscopy observations of BALB/c mice infected with 1 × 10^6^ oocytes of *C. muris* (**a**) and with 3 × 10^6^ of *C. muris* (**b**). *C. muris* adhered to the surfaces of epithelial cells in the glandular part of the gastric mucosa (**a**, **b**, 5000×) (arrows). The surfaces of the epithelial cells were swollen and disordered. The integrity of the mucosal layer of the gastric glandular had been damaged, and many cilia had fallen off or atrophied

The SEM study showed that *Cryptosporidium* adhered to the surfaces of epithelial cells in the glandular part of the gastric mucosa (Fig. 6). The gastric surface of the glandular part was clearly deformed due to the strong pathological changes; the surfaces of epithelial cells were swollen and disordered and filled with necrotic material. A large number of *Cryptosporidium* could be found attached to the superficial epithelium outside the gastric pits.

## Discussion

*Cryptosporidium* infection in camels has become an increasingly important source of zoonotic disease transmission to humans, especially in communities with poor sanitation and inadequate medical facilities [30]. In recent years, there have been many reports worldwide of camel infections caused by different *Cryptosporidium* species, including *C. parvum*, *C. hominis*, *C. andersoni*, *C. occultus*, *C. bovis*, *C. ubiquitum*, *C. muris*, *Cryptosporidium* rat genotype IV and camel genotypes [30–34]. In China, *C. parvum*, *C. hominis*, *C. ubiquitum*, *C. occultus*, *C. bovis* and *C. andersoni* have been found to be infecting camels [25, 34, 35]. In this study, we first reported a *C. muris* isolate from Bactrian camels by sequence analyses and then verified its identity through infectivity and parasitic sites* via* experimental infection of laboratory animals.

Previous studies have shown that *C. muris* oocysts are ellipsoidal and lack sporocysts. The size of the oocysts from the strain isolated from camels in our study are similar to that reported previously for *C. muris* (7 × 5 μm) [15]. In the phylogenetic analyses of the SSU rRNA, actin and COWP genes, the *Cryptosporidium* isolates formed a cluster with *C. muris*. DNA sequencing showed that the camel *C. muris* isolates at the COWP and actin loci had 100% identity with the *C. muris* RN66 isolate and had 96% identify with the camel and human *C. muris* isolates at the SSU rRNA loci. Genetically, the *Cryptosporidium* isolates derived from the Bactrian camels in China belong to *C. muris*. Interestingly, the *C. muris* isolate formed a new MLST subtype: M13, M4, M1, M5. The present *C. muris* MLST subtype clearly differs from previous multilocus sequences based on the MS1, MS2, MS3, and MS16 loci that have been found in other *C. muris* isolate subtypes: a camel-derived subtype (M5, M4, M2, M3) from Egypt [5], a Bactrian camel-derived *C. muris* subtype (M1, M1, M4, M5) from the Czech Republic [5], an ostrich-derived *C. muris* subtype (M5, M4, M6, M4) from China [25] and a human-derived *C. muris* subtype (M1, M2, M4, M5) from Peru [36]. These differences may reflect co-evolution of hosts and parasites, which led to different biological characteristics [5]. This is the first report of *C. muris* in Bactrian camels in China, although *C. muris* spp. have been reported in Bactrian camels in other countries [33].

In our study, no oocysts were observed in control mice, and *Cryptosporidium* oocysts were found in the feces of experimental mice inoculated with oocysts in both treatment groups. Infected mice began to shed small numbers of oocysts on 7–8 dpi, with two peaks in infection intensity during the exposure period. The oocysts lasted 27 days, consistent with previous studies [16, 28, 37], but different from the study of *C. muris* CB03 isolated from BALB/c mice infected with *Camelus bactrianus* [38]. The difference in the prepatent and patent periods may depend on the specific *Cryptosporidium* species and experimental animals [20]. The infected mice did not show diarrhea in our study, which is in agreement with the results reported by Qi et al. [16], who inoculated 10^6^ oocysts of *C. muris* isolated from ostriches into BALB/c mice and Mongolian gerbils, and the study by Anderson [39], who inoculated oocysts of *C. muris* isolated from camels. The mice in different infection dose groups showed no clinical symptoms. The prepatent period advanced as the infective dose increased, and OPG also increased, similar to the previously observed pattern in mice infected with *C. muris* RN66 [40].

In this study, histological sections and SEM studies were used to observe the developmental stages of *Cryptosporidium* in the gastric mucosa epithelium. As expected, a large number of *Cryptosporidium* were found only in the gastric glandular sections, consistent with the colonization of mouse- and camel-derived *C. muris* [39, 41]. While the stomach is composed of non-glandular and glandular sections, the endogenous developmental stage of *C. muris* is limited to the glandular part, a pattern that has previously been observed in mice infected with *C. muris* RN66 [40]. Observed histopathological changes showed the presence of gastric pits filled with *Cryptosporidium* at numerous developmental stages. Morever, there was an extension of the gastric longitudinal fold. SEM observations showed that *C. muris* adhered to the surfaces of the epithelial cells of the gastric mucosa gland; the cells were clearly deformed, and the surfaces of the epithelial cells were swollen and disordered, findings that have previously been observed in BALB/c mice infected with *C. muris* CB03 [38]. This study demonstrated that the parasitic site of *C. muris* is the gastric glandular section, consistent with previous results [5, 16, 41].

## Conclusions

In conclusion, we identified the species of *Cryptosporidium* from camels as *C. muris* through molecular identification and animal experiments. The clinical status and parasitic site of camel-derived *C. muris* were similar to those of mouse-derived *C. muris*, but different in the pattern of oocyst excretion, suggesting that the parasite presents a zoonotic risk. We established an animal infection model of *C. muris* and studied the parasitological and histopathological characteristics of infected mice. However, how the parasite infects camels, the parasitized sites in camels and the mechanism of zoonotic transfer need further study.

## Data Availability

Data supporting the conclusions of this article are included within the article. Nucleotide sequences were deposited in the GenBank database under the accession numbers MT708027, and MT721871 to MT721876.

## Abbreviations

COWP: *Cryptosporidium* oocyst wall protein
MS: Minisatellite
OPG: Oocysts per gram
SSU rRNA: Small-subunit ribosomal RNA

## References

[1] Xiao L (2010). Molecular epidemiology of cryptosporidiosis: an update. Exp Parasitol.

[2] Ryan U, Fayer R, Xiao L (2014). *Cryptosporidium* species in humans and animals: current understanding and research needs. Parasitology.

[3] Jeková J, Limpouchová Z, Prediger J, Holubová N, Sak B, Konečný R (2021). *Cryptosporidium myocastoris* n. sp. (Apicomplexa: Cryptosporidiidae), the species adapted to the nutria (*Myocastor coypus*). Microorganisms.

[4] Thompson RCA, Ash A (2016). Molecular epidemiology of *Giardia* and *Cryptosporidium* infections. Infect Genet Evol.

[5] Feng Y, Yang W, Ryan U, Zhang L, Kvác M, Koudela B (2011). Development of a multilocus sequence tool for typing *Cryptosporidium muris* and *Cryptosporidium andersoni*. J Clin Microbiol.

[6] Xiao L, Feng Y (2017). Molecular epidemiologic tools for waterborne pathogens *Cryptosporidium* spp. and *Giardia duodenalis*. Food Waterborne Parasitol.

[7] Tyzzer EE (1907). A sporozoan found in the peptic glands of the common mouse. Exp Biol Med.

[8] Čondlová Š, Horčičková M, Havrdová N, Sak B, Hlásková L, Perec-Matysiak A (2019). Diversity of *Cryptosporidium* spp. in *Apodemus* spp. in Europe. Eur J Protistol.

[9] Zhao Z, Wang R, Zhao W, Qi M, Zhao J, Zhang L (2015). Genotyping and subtyping of *Giardia* and *Cryptosporidium* isolates from commensal rodents in China. Parasitology.

[10] Li J, Dan X, Zhu K, Li N, Guo Y, Zheng Z (2019). Genetic characterization of *Cryptosporidium* spp. and *Giardia duodenalis* in dogs and cats in Guangdong, China. Parasites Vectors.

[11] Chen L, Hu S, Jiang W, Zhao J, Li N, Guo Y (2019). *Cryptosporidium parvum* and *Cryptosporidium hominis* subtypes in crab-eating macaques. Parasites Vectors.

[12] Huang J, Zhang Z, Zhang Y, Yang Y, Zhao J, Wang R (2018). Prevalence and molecular characterization of *Cryptosporidium* spp. and *Giardia duodenalis* in deer in Henan and Jilin, China. Parasites Vectors.

[13] Wagnerová P, Sak B, McEvoy J, Rost M, Matysiak AP, Ježková J (2015). Genetic diversity of *Cryptosporidium* spp. including novel identification of the *Cryptosporidium muris* and *Cryptosporidium tyzzeri* in horses in the Czech Republic and Poland. Parasitol Res.

[14] Xiao L, Fayer R, Ryan U, Upton SJ (2004). *Cryptosporidium* taxonomy: recent advances and implications for public health. Clin Microbiol Rev.

[15] Tyzzer EE (1910). An extracellular *Coccidium*, *Cryptosporidium Muris* (Gen. Et. Sp. Nov.), of the gastric glands of the common mouse. J Med Res.

[16] Qi M, Huang L, Wang R, Xiao L, Xu L, Li J (2014). Natural infection of *Cryptosporidium muris* in ostriches (*Struthio camelus*). Vet Parasitol.

[17] Arrowood MJ, Sterling CR (1987). Isolation of *Cryptosporidium* oocysts and sporozoites using discontinuous sucrose and isopycnic Percoll gradients. J Parasitol.

[18] Bukhari Z, Smith HV (1995). Effect of three concentration techniques on viability of *Cryptosporidium parvum* oocysts recovered from bovine feces. J Clin Microbiol.

[19] Arrowood MJ, Donaldson K (1996). Improved purification methods for calf-derived *Cryptosporidium parvum* oocysts using discontinuous sucrose and cesium chloride gradients. J Eukaryot Microbiol.

[20] Cui Z, Song D, Qi M, Zhang S, Wang R, Jian F (2018). Revisiting the infectivity and pathogenicity of *Cryptosporidium avium* provides new information on parasitic sites within the host. Parasites Vectors.

[21] Xiao L, Morgan UM, Limor J, Escalante A, Arrowood M, Shulaw W (1999). Genetic diversity within *Cryptosporidium parvum* and related *Cryptosporidium* species. Appl Environ Microbiol.

[22] Xiao L, Bern C, Limor J, Sulaiman I, Roberts J, Checkley W (2001). Identification of 5 types of *Cryptosporidium* parasites in children in Lima. Peru J Infect Dis.

[23] Ng J, Pavlasek I, Ryan U (2006). Identification of novel *Cryptosporidium* genotypes from avian hosts. Appl Environ Microbiol.

[24] Amer S, Honma H, Ikarashi M, Tada C, Fukuda Y, Suyama Y (2010). *Cryptosporidium* genotypes and subtypes in dairy calves in Egypt. Vet Parasitol.

[25] Wang R, Jian F, Zhang L, Ning C, Liu A, Zhao J (2012). Multilocus sequence subtyping and genetic structure of *Cryptosporidium muris* and *Cryptosporidium andersoni*. PLoS ONE.

[26] Yuan L, Yan W, Wang T, Qian W, Ding K, Zhang L (2014). Effects of different inoculation routes on the parasitic sites of *Cryptosporidium baileyi* infection in chickens. Exp Parasitol.

[27] Valigurová A, Jirků M, Koudela B, Gelnar M, Modrý D, Slapeta J (2008). Cryptosporidia: epicellular parasites embraced by the host cell membrane. Int J Parasitol.

[28] Cui Z, Dong H, Wang R, Jian F, Zhang S, Ning C (2018). A canine model of experimental infection with *Cryptosporidium canis*. Exp Parasitol.

[29] Thompson JD, Gibson TJ, Plewniak F, Jeanmougin F, Higgins DG (1997). The CLUSTAL_X windows interface: flexible strategies for multiple sequence alignment aided by quality analysis tools. Nucleic Acids Res.

[30] Sazmand A, Joachim A, Otranto D (2019). Zoonotic parasites of dromedary camels: so important, so ignored. Parasites Vectors.

[31] Zahedi A, Lee GKC, Greay TL, Walsh AL, Blignaut DJC, Ryan UM (2018). First report of *Cryptosporidium parvum* in a dromedary camel calf from Western Australia. Acta Parasitol.

[32] El-Alfy ES, Abu-Elwafa S, Abbas I, Al-Araby M, Al-Kappany Y, Umeda K (2019). Molecular screening approach to identify protozoan and trichostrongylid parasites infecting one-humped camels (*Camelus dromedarius*). Acta Trop.

[33] Kvác M, Sak B, Kvetonová D, Ditrich O, Hofmannová L, Modrý D (2008). Infectivity, pathogenicity, and genetic characteristics of mammalian gastric *Cryptosporidium* spp. in domestic ruminants. Vet Parasitol.

[34] Cao Y, Cui Z, Zhou Q, Jing B, Xu C, Wang T (2020). Genetic diversity of *Cryptosporidium* in Bactrian Camels (*Camelus bactrianus*) in Xinjiang, Northwestern China. Pathogens.

[35] Zhang Q, Zhang Z, Ai S, Wang X, Zhang R, Duan Z (2019). *Cryptosporidium* spp., *Enterocytozoon bieneusi*, and *Giardia duodenalis* from animal sources in the Qinghai-Tibetan Plateau Area (QTPA) in China. Comp Immunol Microbiol Infect Dis.

[36] Palmer CJ, Xiao L, Terashima A, Guerra H, Gotuzzo E, Saldías G (2003). *Cryptosporidium muris*, a rodent pathogen, recovered from a human in Perú. Emerg Infect Dis.

[37] Del Coco VF, Córdoba MA, Sidoti A, Santín M, Drut R, Basualdo JA (2012). Experimental infection with *Cryptosporidium parvum* IIaA21G1R1 subtype in immunosuppressed mice. Vet Parasitol.

[38] Jalovecká M, Sak B, Kváč M, Květoňová D, Kučerová Z, Salát J (2010). Activation of protective cell-mediated immune response in gastric mucosa during *Cryptosporidium muris* infection and re-infection in immunocompetent mice. Parasitol Res.

[39] Anderson BC (1991). Experimental infection in mice of *Cryptosporidium muris* isolated from a camel. J Protozool.

[40] Taylor MA, Marshall RN, Green JA, Catchpole J (1999). The pathogenesis of experimental infections of *Cryptosporidium muris* (strain RN66) in outbred nude mice. Vet Parasitol.

[41] Melicherová J, Ilgová J, Kváč M, Sak B, Koudela B, Valigurová A (2014). Life cycle of *Cryptosporidium muris* in two rodents with different responses to parasitization. Parasitology.

